# Supplementary material for: Splice-Junction-Based Mapping of Alternative Isoforms in the Human Proteome
Source: Cell Rep. Author manuscript; Available in PMC 2020 Jan 15. (PMC6961840; doi:10.1016/j.celrep.2019.11.026)
Supplement: 2 [file NIHMS1546469-supplement-2.zip › DF1/PXD006675/RightCoronaryArtery/RightCoronaryArtery_2_CLTCL1_LQEHFQLQNLGINPANIGFSTLTMESDK.pdf]

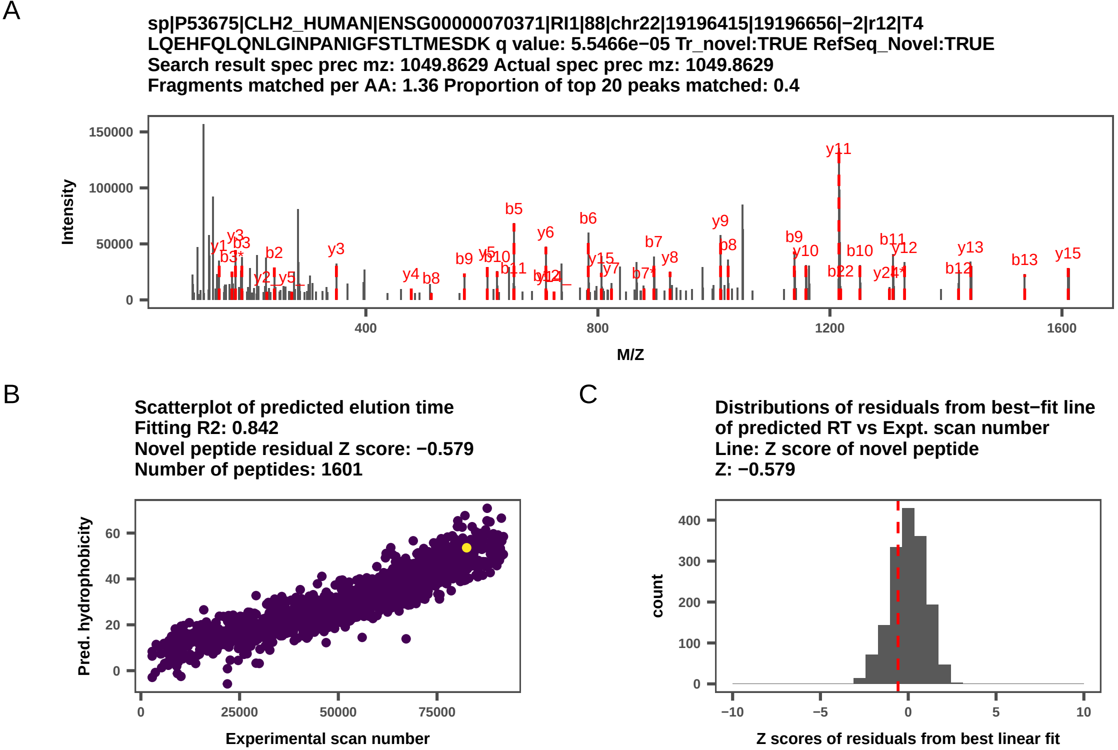
A
sp|P53675|CLH2_HUMAN|ENSG00000070371|RI1|88|chr22|19196415|19196656|−2|r12|T4
LQEHFQLQNLGINPANIGFSTLTMESDK q value: 5.5466e−05 Tr_novel:TRUE RefSeq_Novel:TRUE
Search result spec prec mz: 1049.8629 Actual spec prec mz: 1049.8629
Fragments matched per AA: 1.36 Proportion of top 20 peaks matched: 0.4
Intensity
150000
100000
50000
0
400
800
1200
1600
M/Z
y11
b5
b6
y6
y9
b7
b9
b11
y13
y15
b13
b8
y8
b10
y10
y12
b12
B
Scatterplot of predicted elution time
Fitting R2: 0.842
Novel peptide residual Z score: −0.579
Number of peptides: 1601
Pred. hydrophobicity
60
40
20
0
0
25000
50000
75000
Experimental scan number
C
Distributions of residuals from best−fit line
of predicted RT vs Expt. scan number
Line: Z score of novel peptide
Z: −0.579
count
400
300
200
100
0
−10
−5
0
5
10
Z scores of residuals from best linear fit
